# Indicators associated with severity and mortality in hospitalized people with HIV: A retrospective cohort

**DOI:** 10.1590/0034-7167-2024-0204

**Published:** 2025-01-13

**Authors:** Jarbas da Silva Ziani, Jenifer Härter, Francielle Liz Monteiro, Camila Biazus Dalcin, Stela Maris de Mello Padoin, Marcelo Ribeiro Primeira, Laís Mara Caetano da Silva Corcini, Cláudia Zamberlan

**Affiliations:** I Universidade Federal de Santa Maria. Santa Maria, Rio Grande do Sul, Brazil; II Universidade Federal do Pampa. Uruguaiana, Rio Grande do Sul, Brazil; III Universidade Franciscana. Santa Maria, Rio Grande do Sul, Brazil; IV University of Dundee. Scotland, United Kingdom

**Keywords:** Acquired Immunodeficiency Syndrome, HIV, Hospitalization, Risk, Nursing, Síndrome de Inmunodeficiencia Adquirida, VIH, Hospitalización, Riesgo, Enfermería

## Abstract

**Objectives:**

to compare the sociodemographic and clinical severity indicators of hospitalized people with HIV in relation to clinical outcomes and urgent hospital admission.

**Methods:**

a retrospective cohort study was conducted with 102 medical records of HIV-infected individuals hospitalized in a hospital in southern Brazil. In addition to descriptive analysis, Fisher’s exact test, Pearson’s Chi-square, and logistic regression were used.

**Results:**

the data showed a significant direct effect on severity indicators in the following variables: male sex (p=0.013), skin color (p=0.023), level of education (p=0.000), urgent admissions (p=0.000), late diagnosis (p=0.001), diabetes mellitus (p=0.001), hypertension (p=0.004), kidney disease (p=0.002), high viral load (p=0.006), CD4+ count below 200 (p=0.005), fever (p=0.016), weight loss (p=0.013), co-infection with hepatitis C (p=0.004), and mortality (p=0.007).

**Conclusions:**

three sociodemographic and thirteen clinical markers were identified as being associated with the risk of clinical deterioration in hospitalized people with HIV.

## RESUMEN

**Objetivos:**

comparar los indicadores sociodemográficos y de gravedad clínica de personas con VIH hospitalizadas, en relación con el desenlace y la admisión urgente en la hospitalización.

**Métodos:**

cohorte retrospectiva, realizada con 102 expedientes médicos de personas infectadas por el VIH hospitalizadas en un hospital en el sur de Brasil. Además del análisis descriptivo, se utilizó la prueba exacta de Fisher, la prueba de Chi-cuadrado de Pearson y la regresión logística.

**Resultados:**

los datos mostraron un efecto directo significativo para los indicadores de gravedad en las siguientes variables: sexo masculino (p=0,013), color de piel (p=0,023), nivel de escolaridad (p=0,000), internaciones urgentes (p=0,000), diagnóstico tardío (p=0,001), diabetes mellitus (p=0,001), hipertensión arterial (p=0,004), enfermedad renal (p=0,002), carga viral elevada (p=0,006), CD4+ menor de 200 (p=0,005), presentar fiebre (p=0,016), pérdida de peso (p=0,013), coinfección por hepatitis C (p=0,004) y mortalidad (p=0,007).

**Conclusiones:**

se identificaron tres marcadores sociodemográficos y trece clínicos asociados con el riesgo de agravamiento clínico en personas hospitalizadas con VIH.

## INTRODUCTION

The Human Immunodeficiency Virus (HIV) has been present in society for four decades and remains a serious global public health issue. In an effort to mitigate this problem, the Paris Declaration established the “95-95-95” target in partnership with the Joint United Nations Programme on HIV/AIDS (UNAIDS), aiming to have 95% of people living with HIV (PLHIV) aware of their serostatus; of those aware, 95% on antiretroviral therapy (ART); and, among those on ART, 95% achieving viral suppression^(1)^. Even more ambitious, in 2023, the global strategy report in response to Acquired Immunodeficiency Syndrome (AIDS) launched a new challenge with the goal of ending AIDS by 2030^(2)^.

However, despite the efforts proposed to end the HIV/AIDS epidemic, it is evident that the world is still far from achieving these goals, as epidemiological and operational indicators continue to show high infection rates. In 2022 alone, approximately 1.3 million people were newly infected with HIV, 9.2 million PLHIV did not have access to ART, and 630,000 people died from AIDS-related illnesses worldwide^(3)^. When analyzing the indicators in the Brazilian context, it is clear that, although the epidemic is considered stable, the epidemiological situation requires attention, as in 2022 alone, 990,000 cases of AIDS were reported, with 267,000 of those not adhering to ART and 13,000 resulting in death^(3)^.

Among the current strategies in response to the HIV/AIDS epidemic, the analysis of factors is relevant, making it essential to understand or identify the reasons why PLHIV are being hospitalized for this health condition. Although there is still no cure for HIV, science has already achieved remarkable results in its treatment. It is known that, with the use of ART, people can have a life expectancy equal to that of a person not infected with HIV, a stark contrast to the 1980s, when the first consequences of the infection emerged, a time when life expectancy was only two years after diagnosis^(4)^. In a study conducted in China, 1,551 individuals diagnosed with HIV between 2008 and 2020 required hospitalization at least once due to the infection or its complications^(5)^. Brazil also reported high hospitalization rates, with 144,671 hospitalizations recorded between 2016 and 2020^(6)^.

Therefore, it is necessary to understand the predictive factors of severity that lead PLHIV to hospitalization, as there is evidence related to male sex, advanced age, co-infections, abandonment of ART, CD4+ T lymphocytes ≤ 200 cells/μl, detectable viral load (VL), and discontinuation of seeking health services for the diagnosis and/or treatment of health conditions associated with HIV infection^(7,8,9)^. Given these findings, it is important to identify these risk factors to assist in the planning of care strategies, reducing hospitalization rates, and, most importantly, decreasing the mortality rate associated with the infection/disease^(10)^.

In light of this, this research is justified by its purpose of promoting reflections on the factors that lead PLHIV to hospitalization due to HIV infection, a situation that, depending on aggravating characteristics, can result in death. Additionally, longitudinal studies like this one aim to contribute to the practice of health professionals, especially nurses who care for these individuals, by guiding better clinical decisions and establishing therapeutic plans to mitigate adversities, thereby promoting greater benefits in the health and disease process of PLHIV.

## OBJECTIVES

To compare the sociodemographic and clinical severity indicators of hospitalized people with HIV related to clinical outcomes and urgent hospital admission.

## METHODS

### Ethical Aspects

The study was conducted in accordance with national and international ethical guidelines and received approval from the Research Ethics Committee of the Universidade Franciscana de Santa Maria, with the approval document attached to this submission. The Informed Consent Form (ICF) was waived because the data were obtained from patient records related to hospitalizations that occurred prior to data collection. Additionally, due to the high mortality context resulting from AIDS complications, it is likely that many of the cases treated involve deaths, hospitalizations, or non-autochthonous cases, making it impractical to obtain the ICF.

### Study Design, Period, and Location

This was a quantitative, retrospective cohort study of a documentary nature. The methodological description of this research was guided by the Checklist for Reporting Results of Internet E-Surveys guidelines and the Strengthening the Reporting of Observational Studies in Epidemiology (STROBE) guidelines^(11)^. The research was conducted at a teaching hospital in southern Brazil, known for its technical quality and serving more than 33 municipalities in the region, with all services fully linked to the Unified Health System (in Portuguese SUS).

### Population: Inclusion and Exclusion Criteria

The population consisted of medical records of individuals diagnosed with HIV infection who were hospitalized over a period of one year and one month (January 1, 2022, to January 31, 2023). The research period was established to mitigate the pandemic effect on data analysis, as the hospital was a reference center for treating COVID-19 patients in 2020 and 2021.

Inclusion criteria were medical records of individuals over 18 years old, with hospital stays of at least 72 hours in a clinical unit. Exclusion criteria included records with more than 80% incomplete data in the system for the study variables. A minimum sample size of 94 medical records of people with HIV was considered, with a 5% margin of error, a 95% confidence interval, and a population characteristic proportion of 0.5.

**Figure 1:**
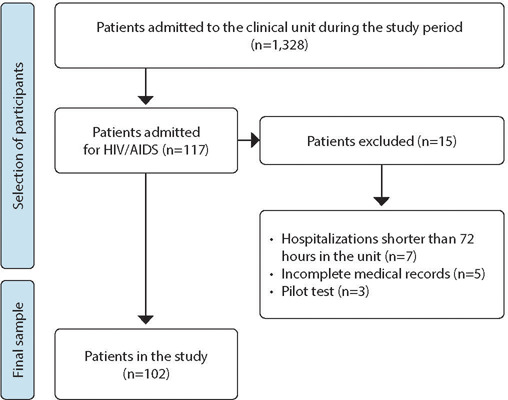
Flowchart of the number of eligible people for the study (n=102), Santa Maria, Rio Grande do Sul, Brazil, 2022-2023

The recruitment process involved reviewing the electronic medical records of participants. To capture eligible records, researchers analyzed all records of individuals admitted during the established period in the unit in question and selected only those whose hospitalization was related to HIV. Additionally, to minimize selection bias, the records were reviewed twice by independent researchers. Thus, the final sample consisted of 102 medical records of people hospitalized for HIV, as described in Figure 1.

### Study Protocol

Data collection occurred between February and June 2023, using an instrument developed by the researchers based on the recommendations of the clinical protocol and therapeutic guidelines for the management of HIV infection in adults from the Ministry of Health^(12)^. Additionally, the instrument was reviewed by five professionals experienced in the HIV field to evaluate the content analysis and semantics of the instrument. After the specialists’evaluation, a pilot test was conducted. Data collection was carried out by the lead researcher and a data collector, both of whom were previously trained.

The questionnaire consisted of sociodemographic questions, such as: sex (male and female), skin color (white and black/mixed-race), education level (less than eight years of schooling and more than eight years of schooling), and vulnerability status (general population, living on the street or in a street situation, drug and/ or alcohol users, and incarcerated individuals).

The clinical variables selected were: reason for hospitalization (HIV/AIDS or other causes), hospital admission type (planned or urgent), time of diagnosis (during hospitalization or prior to hospitalization), co-infection (tuberculosis/HIV), comorbidities (Diabetes Mellitus – DM, Hypertension – HTN, Chronic Kidney Disease – CKD), and absence of comorbidities, diagnostic method (Elisa I and II tests and rapid test), and viral load (undetectable, low, and high). It is important to note that, to categorize viral load as low or high, the Ministry of Health’s guidelines were followed, with low viral load considered as those with results below 200 copies/µl, and high as results above 1000 copies/µl. Information was also collected regarding CD4+ lymphocytes (CD4 count greater than 201 copies/µl and CD4 count less than 200 copies/µl) and initiation of ART (yes and no).

Regarding symptomatology, the dichotomous variables (yes/ no) used were: chronic diarrhea, fever greater than or equal to 38°C for a period greater than or equal to one month, weight loss greater than 10%, asthenia, persistent dermatitis, persistent cough, and lymphadenopathy. Concerning serology, the dichotomous variables (reactive/non-reactive) were: HBsAG, antibodies for Hepatitis A (anti-HVA), anti-HCV, and Venereal Disease Research Laboratory (VDRL).

### Analysis of Results and Statistics

A database was created using Microsoft Excel®, version 10, with dual data entry followed by validation to minimize measurement bias. After validating the spreadsheet and correcting the identified errors, the data were transferred to the final database in the Statistical Package for the Social Sciences (SPSS), version 24.

For data analysis, descriptive statistics were applied, using categorical variables: absolute frequency (n) and relative frequency (%), mean, minimum, maximum, and standard deviation (SD) (±). Additionally, Pearson’s Chi-Square and Fisher’s Exact Test were used for association tests, considering a 5% significance level and a 95% confidence interval (CI95%). To verify the factors associated with severity indicators and clinical outcomes, two logistic regression analyses were performed. The first analysis had the patient’s admission type as the dependent variable, classified as either planned or urgent. For clarity, planned admission refers to those individuals admitted to investigate an HIV infection diagnosis or other clinical health conditions, but who already had a previous HIV diagnosis. Urgent admission, on the other hand, refers to individuals who were admitted from the emergency unit in a severe condition.

In the second analysis, the dependent variable was the clinical outcome, categorized as hospital discharge or death. It should be noted that each variable was tested individually, and those with a p-value <0.05 were included in the initial multiple model. To find the best model fit, the Forward Stepwise methodology was used, and a significance level of 5% and a 95% confidence interval were adopted for inclusion in the final model.

## RESULTS

A total of 117 hospital admissions due to HIV were recorded from January 1, 2022, to January 31, 2023. However, after applying the exclusion criteria, the study sample consisted of 102 participants. Regarding the sociodemographic profile of the participants, the majority were male at birth (58.82%), with an average age of 43.28 years, ranging from 18 to 78 years (SD±12). It was observed that 56.86% were Black/mixed-race, and 59.8% had more than eight years of education.

To better understand the data, Table 1 presents the characterization of individuals hospitalized for HIV and the relationship between variables and outcomes, specifically hospital discharge and death.

**Table 1:** Characterization of individuals hospitalized for HIV and the relationship between variables and outcomes (hospital discharge and death), Santa Maria, Rio Grande do Sul, Brazil, 2022-2023, (n=102)

Variable	Hospital Discharge 74 (72.5%)	Death 28 (27.5%)	Total 102 (100%)	*p* value*
Sex				0.013**
Male	38 (51.4%)	22 (78.6%)	60 (58.82%)
Female	36 (48.6%)	6 (21.4%)	42 (41.18%)
Race				0.023**
White	37 (50%)	7 (25%)	44 (43.14%)
Black/mixed-race	37 (50%)	21 (75%)	58 (56.86)
Education				0.000**
< 8 years	20 (27%)	21 (75%)	41 (40.2%)
8 years or more	54 (73%)	7 (25%)	61 (59.8%)
Admission Type				0.000***
Planned	47 (63.5%)	1 (3.6%)	48 (47.06%)
Urgent	27 (36.5%)	27 (96.4%)	56 (52.94%)
Time of Diagnosis				0.001**
Prior to hospitalization	42 (56.8%)	6 (21.4%)	48 (47.06%)
During hospitalization	32 (43.2%)	22 (78.6%)	54 (52.94%)
Viral Load				0.006***
Undetectable	9 (12.2%)	0 (0%)	9 (8.82%)
Low	4 (5.4%)	0 (0%)	4 (3.92%)
High	61 (82.4%)	28 (100%)	89 (87.25%)
CD4+				0.005***
< 200 copies/µl	48 (64.9%)	26 (92.9%)	74 (72.55%)
201 copies/µl	26 (35.1%)	2 (7.1%)	28 (27.45%)
Initiation of ART				0.000**
Yes	64 (86.5%)	11 (39.3%)	75 (73.53%)
No	10 (13.5%)	17 (60.7l%)	27 (16.76%)

* – p<0.05; ** – Pearson’s Chi-square Test; *** – Fisher’s Exact Test.

Statistical associations were identified (Table 1) between the patient’s outcome in the unit (hospital discharge or death) and the variables sex (p=0.013), race (p=0.023), level of education (p=0.000), type of patient admission to the unit (p=0.000), timing of HIV diagnosis (p=0.001), viral load result (p=0.006), CD4+ count (p=0.005), and timing of ART initiation (p=0.000). Table 2 shows the clinical conditions of PLHIV during hospitalization. Statistical associations were found for the variables DM (p=0.001), HA (p=0.004), CKD (p=0.002), other comorbidities (p=0.028), fever (p=0.016), weight loss (p=0.013), and asthenia (p=0.039).

**Table 2:** Description of comorbidities and symptomatology of individuals hospitalized for HIV and the relationship between variables and outcomes (hospital discharge and death), Santa Maria, Rio Grande do Sul, Brazil, 2022-2023, (n=102)

Variable	Hospital Discharge 74 (72.5%)	Death 28 (27.5%)	Total 102 (100%)	*p* value*
TB/HIV Co-infection				0.357
Yes	22 (29.7%)	11 (39.3%)	33 (32.35%)
No	52 (70.3%)	17 (60.7%)	69 (67.66%)
Diabetes Mellitus				0.001**
Yes	21 (28.4%)	18 (64.3%)	39 (38.24%)
No	53 (71.6%)	10 (35.7%)	63 (63.76%)
Hypertension				0.004**
Yes	24 (32.4%)	18 (64.3%)	42 (41.18%)
No	50 (67.6%)	10 (35.7%)	60 (58.82%)
Chronic Kidney Disease				0.002**
Yes	68 (91.9%)	19 (67.9%)	87 (85.29%)
No	6 (8.1%)	9 (32.1%)	15 (14.71%)
Other Comorbidities				0.028***
Yes	24 (32.4%)	4 (14.3%)	28 (27.45%)
No	50 (67.6%)	24 (85.7%)	74 (72.55%)
Chronic Diarrhea				0.436
Yes	44 (59.5%)	19 (67.9%)	63 (61.76%)
No	30 (40.5%)	9 (32.1%)	39 (38.24%)
Fever				0.016***
Yes	45 (60.8%)	24 (85.7%)	69 (67.65%)
No	29 (39.2%)	4 (14.3%)	33 (32.35%)
Weight Loss				0.013**
Yes	38 (51.4%)	22 (78.6%)	60 (58.82%)
No	36 (48.6%)	6 (21.4%)	42 (41.18%)
Asthenia				0.039**
Yes	36 (48.6%)	20 (71.4%)	56 (54.9%)
No	38 (51.4%)	8 (28.6%)	46 (45.1%)
Persistent Dermatitis				0.092
Yes	19 (25.7%)	12 (42.9%)	31 (30.39%)
No	55 (74.3%)	16 (57.1%)	71 (69.61%)
Cough				0.277
Yes	53 (71.6%)	23 (82.1%)	76 (74.5%)
No	21 (28.4%)	5 (17.9%)	26 (25.5%)
Lymphadenopathy				0.875
Yes	41 (55.4%)	16 (57.1%)	57 (55.88%)
No	33 (44.6%)	12 (42.9%)	45 (44.12%)

* – p<0.05; ** – Pearson’s Chi-square Test; *** – Fisher’s Exact Test.

Regarding the prevalence of serologies performed among the investigated participants, the association between the anti-HCV result and death is noteworthy (p<0.004). Of the 102 participants, 81 (79.4%) were non-reactive, while 21 (20.6%) were reactive, with 11 (39.3%) of the reactive group progressing to death. Additionally, 98 (96.1%) presented a non-reactive result for anti-HBS, while four (3.9%) were reactive (p<0.209). Among the participants, 82 (80.4%) had a non-reactive anti-HVA result and 20 (19.6%) were reactive (p<0.399). In the VDRL test, 59 (57.84%) tested non-reactive for syphilis, while 43 (42.16%) were reactive (p<0.059). Regarding the diagnostic method for HIV, 90 (88.24%) were diagnosed using the Elisa I and II tests, while 12 (11.76%) were diagnosed using the rapid test (p<0.672).

In Table 3, it is observed that male participants had a 14 times higher risk of urgent admission compared to women. Additionally, white participants and those without a diagnosis of DM presented protective factors. Moreover, it was found that participants admitted urgently had up to four times the likelihood of death.

**Table 3:** Planned and urgent admission associated with the characteristics of individuals hospitalized for HIV, Santa Maria, Rio Grande do Sul, Brazil, 2022-2023, (n=102)

Variables	Crude Odds	95% CI*	Adjusted Odds	95% CI	*p* value**
Sex					0.000
Male	12.67	[0.73-1.12]	14.52	[1.41-1.74]
Female	1		1		
Skin Color					0.001
White	0.30	[0.69-0.91]	0.10	[0.12-0.39]
Black/Mixed-race	1		1		
Diabetes Mellitus					0.143
No	0.95	[0.65-1.14]	0.38	[0.10-0.13]
Yes	1		1		
Kidney Disease					0.009
No	3.87	[1.47-1.86]	3.02	[1.01-1.37]
Yes	1		1		
Clinical Outcome					0.007
Hospital discharge	3.04	[1.12-1.39]	4.48	[1.05-1.42]
Death	1		1		

*CI – Confidence Interval; ** – p<0.05.

Table 4 presents the best model from the logistic regression analysis, composed of five variables based on the outcome between hospital discharge and death. It was found that individuals with white skin color had a protective factor, with four times lower odds of death compared to Black/mixed-race individuals. Regarding education, those with more than eight years of schooling had 13 times lower odds of death. Not having kidney disease was a protective factor, with a 10 times lower risk, and not being diabetic resulted in a seven times lower risk of death.

**Table 4:** Sociodemographic and clinical characteristics associated with hospital discharge and death among individuals hospitalized for HIV, Santa Maria, Rio Grande do Sul, Brazil, 2022-2023, (n=102)

Variables	Crude Odds	95% CI*	Adjusted Odds	95% CI	*p* value**
Skin Color					0.024
White	4.74	[0.67-5.06]	4.54	[1.21-16.99]
Black/Mixed-race	1		1		
Level of Education					0.000
More than eight years	2.59	[0.67-14.95]	13.27	[0.20-0.27]
More than eight years	1		1		
Diabetes Mellitus					0.008
No	1.65	[0.62-6.93]	7.19	[0.05-0.65]
Yes	1		1		
Hypertension					0.055
No	1.19	[0.62-3.67]	0.30	[0.08-1.02]
Yes	1		1		
Kidney Disease					0.015
No	1.96	[0.81-5.86]	10.14	[1.02-3.68]
Yes	1		1		

*CI – Confidence Interval; ** – p<0.05.

## DISCUSSION

The present study aimed to compare hospital severity indicators in people with HIV related to clinical outcomes and the type of hospital admission. The results showed that sex was a significant factor during the hospitalization period, as male sex was more prone to death and had a higher likelihood of urgent admission. This finding was corroborated by a global American study, which found that hospitalized men exhibited conditions up to nine times more severe and had a 78% higher mortality rate compared to women^(13)^.

Thus, it is clear that greater investment is needed in campaigns that promote strategies to prevent clinical deterioration in men’s health. A study conducted in Uganda found that HIV infection cases in men are associated with greater infection severity, as men are more likely to have difficulty admitting they have a health problem, seeking support, or remaining engaged in treatment^(14)^.

Similarly, it was noted that non-white skin color and lower levels of education were associated with clinical outcomes and urgent admission. In both variables, the risk of death and the likelihood of urgent admission were significant indicators of severity, leading to unfavorable outcomes. This aligns with other studies indicating that Black and mixed-race individuals have less access to healthcare services, experience delays in care, and consequently, have worse clinical prognoses^(15)^. Furthermore, education level is a predictive factor for lower survival, particularly among individuals with lower educational attainment and those from Black/mixed-race groups^(15)^.

Regarding the mode of admission for these individuals, the data raise concerns, as it was identified that 96.4% of those who were urgently hospitalized progressed to death. Improving early diagnosis of HIV is essential to reducing morbidity and mortality related to the infection. A study conducted in Georgia linked mortality in patients hospitalized in severe conditions due to AIDS to a 32 times higher risk when associated with irregular care markers in PLHIV, as these individuals often lose follow-up and return to care only when the disease has progressed to the point of requiring hospitalization^(16)^.

Regarding the high mortality related to hospitalizations, a study conducted in Spain found that 64.3% of deaths were associated with people hospitalized with HIV who had the same demographic characteristics as those in the current study^(17)^. Therefore, it is crucial to involve Primary Health Care (PHC) in investing in combined prevention strategies and expanding access to care for PLHIV, particularly in terms of early diagnosis. In this way, the importance of new intersectoral public policy structures is highlighted, which can positively impact the management of the condition, minimize unfavorable outcomes, and reduce public costs associated with hospitalizations for this health condition.

The results indicated a high number of participants who died due to late diagnosis, meaning the infection was identified during hospitalization when patients were already in a severe clinical condition. This finding aligns with a German study that demonstrated late diagnosis is associated with increased morbidity and mortality^(18)^.

Given these aspects, the fundamental role of nursing in managing care for PLHIV is highlighted, particularly through actions that contribute to advances in public policies, practices, and research in the contexts where these individuals are situated. This approach ensures direct and continuous care with the implementation of interventions that meet the specific needs of these patients. Additionally, researchers have emphasized the importance of early interventions, as they reduce the likelihood of hospitalization due to HIV-related complications by 85%^(19)^.

An American study underscores the importance of health institutions remaining committed to implementing actions that encourage approaches focused on overcoming barriers that prevent PLHIV from actively participating in their care plans and managing the risk factors for HIV-related morbidity^(20)^. There is evidence that the involvement of a multidisciplinary team in the care of HIV patients offers a protective factor up to 37 times greater than in units without such a team, as multidisciplinary follow-up promotes greater patient engagement and commitment to viral suppression care^(21)^. Therefore, as highlighted by these aspects, the hospital environment should also be understood as a conducive setting for engaging patients in their health and disease process.

Consequently, among the factors associated with the continuous care cascade for PLHIV—related to diagnosis, treatment, and viral suppression—challenges remain in ensuring comprehensive care for these individuals. Scientific evidence points out that treatment offers benefits to quality of life, such as increased life expectancy, reduced virus transmission, viral suppression, more robust immune reconstitution, better clinical outcomes, and reduced hospitalization and mortality rates^(22,23)^.

This is also corroborated by this study, where significant statistical associations were found between people not using ART (p=0.000), those with high viral load (p=0.006), and those with a CD4+ count below 200 copies/µl (p=0.005), with urgent hospitalizations and death. Thus, it is clear that the results reaffirm the need to implement innovative care technologies, ensure continuous government commitment, involve civil society, and, most importantly, establish a collaborative and multidisciplinary approach to the care of PLHIV^(24)^.

A Brazilian study conducted with 550 PLHIV demonstrated a high prevalence of conditions also noted among the participants, such as hypertension (HA) (17.89%), diabetes mellitus (DM) (7.51%), and chronic kidney disease (CKD) (4.83%)^(25)^, although at levels lower than those found in this research. It is important to highlight that, when analyzing chronic non-communicable diseases (NCDs), statistical associations were identified that revealed that PLHIV hospitalized during the study period with HA, DM, and CKD were those with the highest risk of death and urgent hospitalization.

In analyzing the factors associated with CKD in PLHIV, a Brazilian case-control study conducted with 85 participants—17 cases and 68 controls—found that CKD is associated with HA, nephrotoxic antiretroviral medications, and patients over 40 years of age^(26)^. This finding was reaffirmed in an American study, which, in addition to the factors mentioned, also identified DM as a risk factor for developing CKD in PLHIV^(27)^.

The relationship between DM and HIV has also been identified by other studies as a risk factor for the clinical worsening of the disease, as it is related to low CD4+ counts, the use of nucleoside reverse transcriptase inhibitors, protease inhibitors, elevated circulating inflammatory markers, and weight gain^(28,29)^. Furthermore, studies indicate that ART has been associated with weight gain due to the restoration of immunity in PLHIV^(28)^. According to the findings, DM remained a risk factor for the participants, as those who did not report having DM had a protective factor up to seven times greater for avoiding death compared to those who reported living with this comorbidity.

Thus, considering the severity of NCDs for PLHIV, there is a need for further research to evaluate the quality of care for this population that may require hospitalization, with a focus on monitoring these comorbidities and producing information, particularly regarding the adequacy, benefits, adverse effects, technology costs, resoluteness, and continuity of care. These reflections are essential, as care for PLHIV should be delivered with quality, empathy, trust, and continuity, considering the barriers these patients face in seeking healthcare services^(30)^.

When exploring the symptoms presented by the participants, it was observed that fever greater than or equal to 38 degrees for a period of one month or more (p=0.016), weight loss exceeding 10% (p=0.013), and asthenia (p=0.039) were markers associated with the severity and death of the study participants. Reinforcing these findings, a cohort study conducted with PLHIV across two continents, Africa and Asia, demonstrated that fever, weight loss, and asthenia are frequent clinical signs and symptoms among participants, particularly those who had worse clinical prognoses of the infection^(31)^. In addition, a study conducted in Germany with the same population emphasizes the importance of healthcare professionals remaining vigilant to increases in fever, as it was primarily associated with patients who progressed to death in the study^(32)^.

It is noted that these signs and symptoms can assist in the diagnosis, monitoring of disease progression, and reduction of health risks for patients in areas where routine laboratory testing is limited due to financial constraints, infrastructure issues, or long wait times for exams. Additionally, the presence of these signs and symptoms can guide the use of rapid tests in diagnosing HIV, thereby improving virus detection^(31)^ and reducing the chances of urgent hospitalizations.

When analyzing HIV and HCV co-infection, it was found that the individuals in the study who presented reactive anti-HCV serological tests were also those who progressed to death due to HIV. This finding indicates that HCV is frequently prevalent in PLHIV^(33,34)^. Moreover, the factors leading to poorer clinical outcomes due to co-infection include the impact that HCV has on the recovery of CD4+ cells in patients undergoing ART. Additionally, HIV infection increases the chances of these patients developing hepatic fibrosis, highlighting the importance of screening for HCV in HIV patients before initiating ART to prevent potential drug interactions^(33)^.

### Study limitations

The number of participants in this study is noteworthy, as even though significant results were achieved, a larger population could influence the outcomes and reduce confidence intervals. Thus, it is important to highlight the need for greater investment in this type of research within the hospital setting. Multicenter studies are suggested to broaden the scope of care for this population and enable comparative analysis with the findings obtained.

### Contributions to the Field

This manuscript provides healthcare professionals, especially those in nursing, with important insights for clinical management in caring for people living with HIV, particularly in the hospital context. Additionally, the study contributes to guiding clinical practice in the organization, planning, and execution of care practices, while also promoting greater autonomy for professionals.

## CONCLUSIONS

Three sociodemographic and thirteen clinical prognostic markers were identified as being significantly associated with the risk of clinical deterioration in people hospitalized with HIV. Regarding sociodemographic variables, male sex was highly associated with death and the risk of urgent hospitalization, while Black/mixed-race skin color showed higher mortality rates, and white skin color was identified as a protective factor. In terms of education level, PLHIV with less than eight years of schooling were at a higher risk of death due to HIV-related complications.

Concerning the clinical characteristics of the disease, urgent hospital admission, diagnosis discovered during hospitalization, and the non-use of ART were directly associated with death. Regarding biological markers, it was found that people hospitalized with high viral loads and CD4+ counts below 200 copies/µl had a higher risk of death and urgent hospitalization, while those with low or undetectable viral loads were more likely to be discharged and admitted in a planned manner.

Similarly, having diabetes mellitus, hypertension, and chronic kidney disease were the comorbidities associated with the highest risk of clinical deterioration due to HIV in the participants. Regarding the signs and symptoms associated with the risk of death and urgent hospitalizations, fever of 38°C or higher for one month or more, weight loss exceeding 10%, and asthenia were among the indicators of the worst prognosis. As for the serological tests performed on the participants, co-infection with HCV was the only indicator that stood out as the most significant risk factor.
