# Supplementary material for: Indicators associated with severity and mortality in hospitalized people with HIV: A retrospective cohort
Source: Rev Bras Enferm. 2025 Jan 13;77(6):e20240204. doi: 10.1590/0034-7167-2024-0204 (PMC11731855; doi:10.1590/0034-7167-2024-0204)
Supplement: Translation

# Translation: Indicadores associados à gravidade e ao óbito em pessoas hospitalizadas por HIV: coorte retrospectiva

**DOI:** 10.1590/0034-7167-2024-0204pt

**Authors:** Jarbas da Silva Ziani, Jenifer Härter, Francielle Liz Monteiro, Camila Biazus Dalcin, Stela Maris de Mello Padoin, Marcelo Ribeiro Primeira, Laís Mara Caetano da Silva Corcini, Cláudia Zamberlan

**Affiliations:** I Universidade Federal de Santa Maria. Santa Maria, Rio Grande do Sul, Brasil; II Universidade Federal do Pampa. Uruguaiana, Rio Grande do Sul, Brasil; III Universidade Franciscana. Santa Maria, Rio Grande do Sul, Brasil; IV University of Dundee. Escócia, Reino Unido

**Keywords:** Síndrome de Imunodeficiência Adquirida, HIV, Hospitalização, Risco, Enfermagem

## Abstract

**Objetivos:**

comparar os indicadores sociodemográficos e de gravidade clínica de pessoas com HIV hospitalizadas, em relação ao desfecho e à admissão urgente na internação hospitalar.

**Métodos:**

coorte retrospectiva, realizada com 102 prontuários de pessoas infectadas pelo HIV hospitalizadas em um hospital no sul do Brasil. Além da análise descritiva, utilizou-se o teste exato de Fisher, Qui-quadrado de Pearson e regressão logística.

**Resultados:**

os dados mostraram efeito direto significativo para indicadores de gravidade nas variáveis: sexo masculino (p=0,013), cor da pele (p=0,023), nível de escolaridade (p=0,000), internações de forma urgente (p=0,000), diagnóstico tardio (p=0,001), diabetes mellitus (p=0,001), hipertensão arterial (p=0,004), doença renal (p=0,002), carga viral elevada (p=0,006), CD4+ menor que 200 (p=0,005), apresentar febre (p=0,016), perda de peso (p=0,013), coinfecção por hepatite C (p=0,004) e óbito (p=0,007).

**Conclusões:**

identificaram-se três marcadores sociodemográficos e treze clínicos associados ao risco de agravamento clínico em pessoas hospitalizadas com HIV.

## INTRODUÇÃO

O Vírus da Imunodeficiência Humana (HIV) está presente na sociedade há quatro décadas e ainda se configura como um grave problema de saúde pública global. No intuito de minimizar essa problemática, a Declaração de Paris pactuou a meta “95-95-95” em parceria com o Programa Conjunto das Nações Unidas sobre HIV/Aids (UNAIDS), que visa ter 95% das pessoas que vivem com HIV (PVHIV) cientes do seu status sorológico; dessas, 95% em uso da terapia antirretroviral (TARV); e, entre as que estão em uso da TARV, 95% em supressão viral^(1)^. Ainda mais ambicioso, em 2023, o relatório de estratégias globais em resposta à Síndrome da Imunodeficiência Adquirida (Aids) lançou um novo desafio com o objetivo de acabar com a Aids até o ano de 2030^(2)^.

Todavia, mesmo diante dos esforços propostos para acabar com a epidemia de HIV e Aids, nota-se que o mundo ainda está distante de alcançar esses objetivos, uma vez que os indicadores epidemiológicos e operacionais demonstram elevadas taxas de infecção. Somente no ano de 2022, cerca de 1,3 milhão de pessoas se infectaram com HIV, sendo que 9,2 milhões de Pessoas Vivendo com HIV (PVHIV) não possuíam acesso à TARV, e 630 mil pessoas morreram por doenças relacionadas à Aids no mundo^(3)^. Ao analisar os indicadores do cenário brasileiro, percebe-se que, apesar de a epidemia ser considerada estável, o quadro epidemiológico requer empenho para sua diminuição, pois, somente no ano de 2022, foram registrados 990 mil casos de Aids, sendo que, desses, 267 mil não estavam aderindo à TARV e 13 mil evoluíram para o óbito^(3)^.

Dentre as estratégias vigentes em resposta à epidemia de HIV e Aids, a análise de fatores é relevante e torna-se imprescindível compreender ou identificar os motivos pelos quais as PVHIV estão sendo hospitalizadas por essa condição de saúde. Embora a infecção pelo HIV ainda não tenha cura, a ciência já promoveu resultados surpreendentes com seu tratamento. Sabe-se que, a partir do uso da TARV, as pessoas podem ter uma expectativa de vida igual à de uma pessoa não infectada pelo HIV, circunstância oposta à dos anos 1980, quando surgiram os primeiros agravos pela infecção, período no qual a expectativa de vida era de apenas dois anos após o diagnóstico^(4)^. Em uma pesquisa realizada na China, 1.551 indivíduos com diagnóstico de HIV entre 2008 e 2020 necessitaram de internação pelo menos uma vez devido à infecção ou às complicações decorrentes dela^(5)^. O Brasil também apresentou índices elevados de internações, visto que, entre 2016 e 2020, registrou-se 144.671 hospitalizações^(6)^.

Dessa forma, torna-se necessário compreender os fatores preditivos de gravidade que levam as PVHIV à internação hospitalar, uma vez que há evidências relacionadas ao sexo masculino, idade avançada, coinfecções, abandono da TARV, linfócitos T CD4+ ≤ 200 células/μl, carga viral (CV) detectável, e desistência da busca pelos serviços de saúde para diagnóstico e/ou tratamento das condições de saúde associadas à infecção pelo HIV^(7,8,9)^. Tendo em vista esses achados, percebe-se a importância da identificação desses fatores de risco para auxiliar no planejamento das estratégias de cuidado, redução dos indicadores de internações e, prioritariamente, na diminuição da taxa de mortalidade associada à infecção/doença^(10)^.

À vista disso, esta pesquisa justifica-se pelo seu propósito de promover reflexões acerca dos fatores que levam as PVHIV à internação hospitalar em decorrência da infecção pelo HIV, fato que, de acordo com características agravantes, pode resultar em óbito. Além disso, estudos longitudinais como este têm a finalidade de contribuir para a prática dos profissionais de saúde, em especial dos enfermeiros que prestam cuidados a essas pessoas, orientando melhores decisões clínicas e o estabelecimento de planos terapêuticos para mitigar adversidades, visando promover mais benefícios no processo de saúde e doença das PVHIV.

## OBJETIVOS

Comparar os indicadores sociodemográficos e de gravidade clínica de pessoas com HIV hospitalizadas relacionadas ao desfecho e a admissão urgente na internação hospitalar.

## MÉTODOS

### Aspectos éticos

O estudo foi conduzido de acordo com as diretrizes éticas nacionais e internacionais, tendo recebido aprovação do Comitê de Ética em Pesquisa da Universidade Franciscana de Santa Maria, cujo parecer está anexado à presente submissão. No que tange, ao Termo de Consentimento Livre e Esclarecido (TCLE) dos participantes o mesmo foi dispensado, tendo em vista que os dados foram obtidos de prontuários de pacientes que estiveram sua internação anterior a coleta dos dados. Ainda em razão do contexto de alta mortalidade decorrente dos agravamentos por aids, é provável que muitos dos casos atendidos se tratem de óbitos, internações ou casos não autóctones, inviabilizando a solicitação do TCLE.

### Desenho, período e local do estudo

Pesquisa quantitativa, coorte retrospectiva de caráter documental. A descrição metodológica dessa pesquisa foi norteada pelas diretrizes do *Checklist for Reporting Results of Internet E-Surveys guidelines and Strengthening the Reporting of Observational Studies in Epidemiology* (STROBE)^(11)^. A pesquisa foi desenvolvida em um hospital escola do sul do Brasil, referência em qualidade técnica e atende mais de 33 municípios da região, sendo seus atendimentos 100% vinculados ao Sistema Único de Saúde.

### População: critérios de inclusão e exclusão

A população foi composta por prontuários de pessoas com diagnóstico de infecção pelo HIV hospitalizadas no período de um ano e um mês (01/janeiro/2022 a 31/janeiro/2023). O período da pesquisa foi estabelecido em virtude de o hospital ter sido referência no atendimento de pessoas com COVID-19 nos anos de 2020 e 2021, buscando, assim, diminuir o efeito pandêmico da análise de dados.

Os critérios de inclusão foram prontuários de pessoas com mais de 18 anos, com internações de no mínimo de 72 horas em unidade clínica. Como critérios de exclusão, considerou-se o fato de os prontuários apresentarem mais de 80% de dados incompletos no sistema para as variáveis da pesquisa. Em relação ao tamanho amostral, considerou-se uma amostra mínima de 94 prontuários de pessoas com HIV, com tolerância de 5% de erro, intervalo de confiança de 95% e proporção da característica de interesse na população de 0,5.

O modo de recrutamento foi realizado mediante a observação dos prontuários eletrônicos dos participantes. Para capturar os prontuários elegíveis, os pesquisadores necessitaram realizar a análise de todos os prontuários das pessoas que foram admitidas no período estabelecido na unidade em questão e selecionar apenas os que tiveram sua internação relacionada ao HIV. Ademais, com a finalidade de minimizar viés de seleção, os prontuários foram analisados duas vezes por pesquisadores independentes. Assim, a amostra final foi composta por 102 prontuários de pessoas hospitalizadas por HIV conforme descrito na Figura 1.

**Figura 1:** Fluxograma do número de pessoas elegíveis para o estudo (n=102), Santa Maria, Rio Grande do Sul, Brasil, 2022-2023

### Protocolo do estudo

A coleta de dados ocorreu no período de fevereiro a junho de 2023, por meio de um instrumento elaborado pelos pesquisadores com base nas recomendações do protocolo clínico e diretrizes terapêuticas para o manejo da infecção pelo HIV em adultos, do Ministério da Saúde^(12)^. Além disso, o instrumento passou pela apreciação de cinco profissionais experientes na temática do HIV, com a finalidade de avaliar a análise de conteúdo e semântica do instrumento. Após a avaliação pelos especialistas, foi realizado o teste piloto. A coleta dos dados foi conduzida pelo autor da pesquisa e por uma coletora de dados, ambos previamente treinados.

O questionário foi composto por questões sociodemográficas, tais como: sexo (masculino e feminino), cor da pele (branca e preta/ parda), escolaridade (menos de oito anos de estudo e mais de oito anos de estudo), e situação de vulnerabilidade (população em geral, vivendo na rua ou em situação de rua, usuários de drogas e/ou álcool, e privado de liberdade).

As variáveis clínicas selecionadas foram: motivo da internação (HIV/Aids ou outras causas), admissão hospitalar (planejada ou urgente), momento do diagnóstico (durante a hospitalização ou anterior à hospitalização), coinfecção (tuberculose/HIV), comorbidades (Diabetes Mellitus – DM, Hipertensão Arterial – HA, Doença Renal Crônica – DRC) e ausência de comorbidades, método diagnóstico (teste de Elisa I e II e teste rápido), e carga viral (indetectável, baixa e alta). Ressalta-se que, para caracterizar as variáveis em carga viral baixa e alta, levou-se em consideração as diretrizes do MS, onde considerou-se carga viral baixa aquelas com resultados inferiores a 200 cópias/µl, e alta, resultados acima de 1000 cópias/µl. Também foram coletadas informações acerca dos linfócitos CD4+ (CD4 maior que 201 cópias/µl e CD4 menor que 200 cópias/µl) e início da TARV (sim e não).

Quanto à sintomatologia, as variáveis dicotômicas (sim/não) utilizadas foram: diarreia crônica, febre maior ou igual a 38°C por período maior ou igual a um mês, perda de peso maior que 10%, astenia, dermatite persistente, tosse persistente e linfadenomegalia. No que diz respeito às sorologias, as variáveis dicotômicas (reagente/não reagente) foram: HBsAG, anticorpos para Hepatite A (anti-HVA), anti-HCV e *Venereal Disease Research Laboratory* (VDRL).

### Análise dos resultados e estatística

Foi desenvolvido um banco de dados no gerenciador de planilhas *Microsoft Excel* ^®^, versão 10, em dupla digitação, seguido de validação para minimizar viés de aferição. Posteriormente a validação da planilha com as correções identificadas, os dados foram transportados para o banco definitivo no *software Statistical Package for the Social Sciences* (SPSS), versão 24.

Para análise dos dados, aplicou-se a estatística descritiva, por meio das variáveis categóricas: frequência absoluta (n) e relativa (%), média, mínima, máxima e desvio padrão (DP) (±). Além dos testes de associação Qui-Quadrado de Pearson e Teste Exato de Fisher, considerando-se um nível de 5% de significância e intervalo de 95% de confiança (IC95%). Com destino a verificar os fatores associados aos indicadores de gravidade e desfecho clínico das pessoas, realizou-se duas análises de regressão logística, sendo a primeira variável dependente à admissão do paciente na unidade, classificada em planejada ou urgência. Para o melhor entendimento, a internação de forma planejada refere-se aquelas pessoas que foram admitidos para investigar o diagnóstico de infecção pelo HIV ou por outras condições clínicas de saúde, porém já com o diagnóstico prévio do HIV. Já a admissão de forma urgente, deu-se pelas pessoas que vieram da unidade de pronto atendimento na forma grave.

Na segunda análise, a variável dependente foi o desfecho clínico, considerada alta hospitalar ou óbito. Reforça-se que cada variável foi testada, individualmente, e as que apresentaram um valor de p <0,05 foram incluídas no modelo múltiplo inicial. Para encontrar o melhor ajuste do modelo utilizou a metodologia de *Forward Stepwise* e adotou-se para permanência no modelo final, nível de significância de 5% e intervalo de 95% de confiança.

## RESULTADOS

Registraram-se 117 admissões hospitalares em decorrência do HIV no período de 1° de janeiro de 2022 a 31 janeiro de 2023, todavia, após critérios de exclusão a amostra do estudo foi composta por 102 participantes. Com relação ao perfil sociodemográfico dos participantes, eram em sua maioria do sexo masculino atribuído ao nascer 58,82%, com média de 43,28 anos, sendo a idade mínima 18 e máxima de 78 (DP±12). Notou-se que 56,86% eram pretos/pardos e 59,8% possuíam mais de oito anos de estudo.

Para melhor entendimento dos dados na Tabela 1, apresenta-se a caracterização das pessoas hospitalizados por HIV e a relação das variáveis com o desfecho entre alta hospitalar e óbito.

**Tabela 1:** Caracterização das pessoas hospitalizados por HIV e a relação das variáveis com o desfecho entre a alta hospitalar e o óbito, Santa Maria, Rio Grande do Sul, Brasil, 2022-2023, (n=102)

Variável	Alta hospitalar 74 (72,5%)	Óbito 28 (27,5%)	Total 102 (100%)	Valor de p*
Sexo				0,013**
Masculino	38 (51,4%)	22 (78,6%)	60 (58,82%)
Feminino	36 (48,6%)	6 (21,4%)	42 (41,18%)
Cor				0,023**
Branca	37 (50%)	7 (25%)	44 (43,14%)
Preta/parda	37 (50%)	21 (75%)	58 (56,86)
Escolaridade				0,000**
< 8 anos	20 (27%)	21 (75%)	41 (40,2%)
8 anos ou mais	54 (73%)	7 (25%)	61 (59,8%)
Forma de admissão				0,000***
Planejada	47 (63,5%)	1 (3,6%)	48 (47,06%)
Urgente	27 (36,5%)	27 (96,4%)	56 (52,94%)
Momento do diagnóstico				0,001**
Anterior à hospitalização	42 (56,8%)	6 (21,4%)	48 (47,06%)
Durante a hospitalização	32 (43,2%)	22 (78,6%)	54 (52,94%)
Carga viral				0,006***
Indetectável	9 (12,2%)	0 (0%)	9 (8,82%)
Baixa	4 (5,4%)	0 (0%)	4 (3,92%)
Alta	61 (82,4%)	28 (100%)	89 (87,25%)
CD4+				0,005***
< 200 cópias/ul	48 (64,9%)	26 (92,9%)	74 (72,55%)
Acima de 201 cópias/ul	26 (35,1%)	2 (7,1%)	28 (27,45%)
Início da TARV				0,000**
Sim	64 (86,5%)	11 (39,3%)	75 (73,53%)
Não	10 (13,5%)	17 (60,7l%)	27 (16,76%)

* – p<0.05; ** – Teste de Qui-quadrado de Person; *** – Teste de Exato de Fisher.

Foi possível identificar associação estatísticas (Tabela 1) entre o desfecho do paciente na unidade (alta hospitalar ou óbito) e as variáveis sexo (p=0,013), cor da pele (p=0,023), nível de escolaridade (p=0,000), forma de admissão do paciente na unidade (p=0,000), momento em que ocorreu o diagnóstico de HIV (p=0,001), resultado da carga viral (p=0,006), CD4+ (p=0,005) e o momento de início da TARV (p=0,000). Na Tabela 2, observam-se as condições clínicas das PVHIV durante a hospitalização. Nota-se que houve associação estatísticas para as variáveis DM (p=0,001), HA (p=0,004), DRC (p=0,002), outras comorbidades (p=0,028), febre (p=0,016), perda de peso (p=0,013), astenia (p=0,039).

**Tabela 2:** Descrição das comorbidades e sintomatologia das pessoas hospitalizadas por HIV e a relação das variáveis com o desfecho entre a alta hospitalar e o óbito, Santa Maria, Rio Grande do Sul, Brasil, 2022-2023, (n=102)

Variável	Alta hospitalar 74 (72,5%)	Óbito 28 (27,5%)	Total 102 (100%)	Valor de p*
Coinfecção TB/HIV				0,357
Sim	22 (29,7%)	11 (39,3%)	33 (32,35%)
Não	52 (70,3%)	17 (60,7%)	69 (67,66%)
Diabetes mellitus				0,001**
Sim	21 (28,4%)	18 (64,3%)	39 (38,24%)
Não	53 (71,6%)	10 (35,7%)	63 (63,76%)
Hipertensão arterial				0,004**
Sim	24 (32,4%)	18 (64,3%)	42 (41,18%)
Não	50 (67,6%)	10 (35,7%)	60 (58,82%)
Doença renal crônica				0,002**
Sim	68 (91,9%)	19 (67,9%)	87 (85,29%)
Não	6 (8,1%)	9 (32,1%)	15 (14,71%)
Outras comorbidades				0,028***
Sim	24 (32,4%)	4 (14,3%)	28 (27,45%)
Não	50 (67,6%)	24 (85,7%)	74 (72,55%)
Diarreia crônica				0,436
Sim	44 (59,5%)	19 (67,9%)	63 (61,76%)
Não	30 (40,5%)	9 (32,1%)	39 (38,24%)
Febre				0,016***
Sim	45 (60,8%)	24 (85,7%)	69 (67,65%)
Não	29 (39,2%)	4 (14,3%)	33 (32,35%)
Perda de peso				0,013**
Sim	38 (51,4%)	22 (78,6%)	60 (58,82%)
Não	36 (48,6%)	6 (21,4%)	42 (41,18%)
Astenia				0,039**
Sim	36 (48,6%)	20 (71,4%)	56 (54,9%)
Não	38 (51,4%)	8 (28,6%)	46 (45,1%)
Dermatite persistente				0,092
Sim	19 (25,7%)	12 (42,9%)	31 (30,39%)
Não	55 (74,3%)	16 (57,1%)	71 (69,61%)
Tosse				0,277
Sim	53 (71,6%)	23 (82,1%)	76 (74,5%)
Não	21 (28,4%)	5 (17,9%)	26 (25,5%)
Linfonodomegalia				0,875
Sim	41 (55,4%)	16 (57,1%)	57 (55,88%)
Não	33 (44,6%)	12 (42,9%)	45 (44,12%)

* – p<0.05; ** – Teste de Qui-quadrado de Person; *** – Teste de Exato de Fisher.

Referente à prevalência das sorologias realizadas entre os participantes investigados, destaca-se a associação entre o resultado do anti-HCV e o óbito (p<0,004). Dos 102 participantes, 81 (79,4%) foram não reagentes, enquanto 21 (20,6%) foram reagentes, sendo que, desses, 11 (39,3%) evoluíram para o óbito. Além disso, 98 (96,1%) apresentaram resultado não reagente para o anti-HBS, enquanto quatro (3,9%) foram reagentes (p<0,209). Entre os participantes, 82 (80,4%) estavam com o anti-HVA não reagente e 20 (19,6%) reagente (p<0,399). No exame de VDRL, 59 (57,84%) testaram não reagente para sífilis e 43 (42,16%) reagente (p<0,059). Quanto ao método diagnóstico para o HIV, 90 (88,24%) tiveram seu diagnóstico pelo teste de Elisa I e II, enquanto 12 (11,76%) foram diagnosticados pelo teste rápido (p<0,672).

Na Tabela 3, observa-se que os participantes do sexo masculino apresentaram um risco 14 vezes maior de internação urgente quando comparados às mulheres. Já os participantes brancos e aqueles sem diagnóstico de DM apresentaram fatores de proteção. Ademais, verifica-se que os participantes que foram admitidos de forma urgente tinham até quatro vezes mais chances de óbito.

**Tabela 3:** Forma de admissão planejada e urgente associada às características das pessoas hospitalizadas por HIV, Santa Maria, Rio Grande do Sul, Brasil, 2022-2023, (n=102)

Variáveis	Odds bruto	*IC* 95%	Odds ajustado	IC 95%	Valor de p**
Sexo					0,000
Masculino	12,67	[0,73-1,12]	14,52	[1,41-1,74]
Feminino	1		1		
Cor da pele					0,001
Branca	0,30	[0,69-0,91]	0,10	[0,12-0,39]
Preta/Parda	1		1		
Diabetes Mellitus					0,143
Não	0,95	[0,65-1,14]	0,38	[0,10-0,13]
Sim	1		1		
Doença Renal					0,009
Não	3,87	[1,47-1,86]	3,02	[1,01-1,37]
Sim	1		1		
Desfecho clínico					0,007
Alta hospitalar	3,04	[1,12-1,39]	4,48	[1,05-1,42]
Óbito	1		1		

*IC – Intervalo de Confiança; ** – p<0.05.

A Tabela 4 indica o melhor modelo da análise de regressão logística composto por cinco variáveis, conforme o desfecho entre a alta hospitalar e o óbito. Asseverou-se que pessoas da cor branca possuíam fator de proteção para o óbito quatro vezes menos chances de óbito em comparação aos pretos/pardos. Quanto à escolaridade, pessoas com mais de oito anos de estudo, apresentaram 13 vezes menos chances de óbito. Não ter doença renal foi um fator de proteção com risco 10 vezes menor e não ser diabético resultou em um risco sete vezes menor para o óbito.

**Tabela 4:** Características sociodemográficas e clínicas associadas à alta hospitalar e ao óbito das pessoas hospitalizadas por HIV, Santa Maria, Rio Grande do Sul, Brasil, 2022-2023, (n=102)

Variáveis	Odds bruto	IC* 95%	Odds ajustado	IC 95%	Valor de p**
Cor da pele					0,024
Branca	4,74	[0,67-5,06]	4,54	[1,21-16,99]
Preta/Parda	1		1		
Nível de escolaridade					0,000
Maior de oito anos	2,59	[0,67-14,95]	13,27	[0,20-0,27]
Maior de oito anos	1		1		
Diabetes Mellitus					0,008
Não	1,65	[0,62-6,93]	7,19	[0,05-0,65]
Sim	1		1		
Hipertensão arterial					0,055
Não	1,19	[0,62-3,67]	0,30	[0,08-1,02]
Sim	1		1		
Doença renal					0,015
Não	1,96	[0,81-5,86]	10,14	[1,02-3,68]
Sim	1		1		

*IC – Intervalo de Confiança; ** – p<0.05.

## DISCUSSÃO

O presente estudo objetivou comparar os indicadores de gravidade hospitalar em pessoas com HIV relacionados ao desfecho clínico e à forma de admissão na internação hospitalar. Os resultados demonstraram que o sexo foi um fator de grande impacto durante o período de hospitalização, uma vez que o sexo masculino foi o mais propenso ao óbito e também o que apresentou maior probabilidade de internação urgente. Esse fato foi reafirmado por um estudo americano realizado em nível global, no qual se constatou que os homens hospitalizados apresentaram quadros até nove vezes mais graves e uma taxa de mortalidade 78% mais elevada em comparação com as mulheres^(13)^.

Assim, é visível a necessidade de maiores investimentos em campanhas que promovam estratégias de prevenção do agravamento clínico para a saúde do público masculino. Um estudo realizado em Uganda afirmou que os casos de infecção por HIV em homens estão relacionados a uma maior gravidade da infecção, uma vez que o sexo masculino possui maior dificuldade em admitir que há um problema em sua saúde, procurar apoio ou permanecer envolvido no tratamento^(14)^.

De forma semelhante, nota-se que as variáveis cor da pele não branca e menor nível de escolaridade apresentaram associações com o desfecho e a admissão urgente. Em ambas as variáveis, o risco para o óbito e a forma de admissão urgente foram indicadores de gravidade determinantes para resultados desfavoráveis. Isso corrobora outros estudos que apontam que pessoas pretas e pardas possuem menor acesso aos serviços de saúde, atraso na assistência e, consequentemente, piores prognósticos clínicos^(15)^. Além disso, verifica-se que a escolaridade é um fator preditivo de menor sobrevida em pessoas com baixa escolaridade, especialmente entre os grupos de cor da pele preta/parda^(15)^.

No que concerne à forma de admissão dessas pessoas na unidade, os dados mostram preocupação, pois foi identificado que 96,4% das pessoas que foram hospitalizadas de forma urgente evoluíram para óbito. Melhorar o diagnóstico precoce do HIV é essencial para diminuir a morbidade e a mortalidade relacionadas à infecção. Um estudo realizado na Geórgia associou a mortalidade em pacientes internados em estado grave por Aids a um risco 32 vezes maior quando atrelada a marcadores irregulares no cuidado às PVHIV, uma vez que essas pessoas perdem o acompanhamento e retornam aos cuidados quando a doença já progrediu para a necessidade de internação^(16)^.

Ainda sobre a alta mortalidade relacionada às internações, um estudo realizado na Espanha apontou que 64,3% dos óbitos estavam relacionados a pessoas hospitalizadas com HIV e com as mesmas características demográficas do estudo em questão^(17)^. Assim, reforça-se a necessidade de envolver a Atenção Primária à Saúde (APS) para investir em estratégias de prevenção combinada, ampliação do acesso ao cuidado às PVHIV, principalmente quanto ao diagnóstico antecipado. Dessa forma, demonstra-se a importância de novas estruturas de políticas públicas de natureza intersetorial, atuando na assistência às PVHIV e, assim, impactando positivamente no manejo do agravo, minimizando os desfechos desfavoráveis e reduzindo os custos públicos com as hospitalizações por essa condição de saúde.

Os resultados indicaram um número elevado de participantes que foram a óbito em decorrência do diagnóstico tardio, ou seja, a infecção foi identificada durante a internação hospitalar, com os pacientes já em situação clínica grave. Esse achado está de acordo com um estudo alemão, que demonstrou que o diagnóstico tardio está associado ao aumento da morbimortalidade^(18)^.

Frente a esses aspectos, destaca-se o papel fundamental que a enfermagem exerce no gerenciamento dos cuidados às PVHIV, principalmente atuando em ações que contribuam para os avanços nas políticas públicas, práticas e investigações dos cenários em que esses indivíduos estão inseridos, proporcionando uma assistência direta e contínua com a implementação de cuidados fidedignos às necessidades desses pacientes. Além disso, os pesquisadores destacaram a importância das ações precoces, visto que elas reduzem em 85% as chances de uma pessoa vir a ser hospitalizada em decorrência de complicações da infecção pelo HIV^(19)^.

Um estudo norte-americano destaca a importância de as instituições de saúde se manterem comprometidas com a implementação de ações que encorajem abordagens centradas nas barreiras que impedem as PVHIV de manter uma participação ativa em seu plano de cuidado e na gestão dos fatores de risco para a morbidade pela infecção do HIV^(20)^. Existem evidências de que a atuação de uma equipe multidisciplinar nos cuidados aos pacientes com HIV promove um fator de proteção até 37 vezes maior do que em unidades que não contam com essa equipe, visto que o acompanhamento multidisciplinar promove maior envolvimento e comprometimento dos pacientes nos cuidados para a supressão viral^(21)^. Dessa maneira, conforme os aspectos supracitados, destaca-se que o ambiente hospitalar também deve ser compreendido como um local propício para engajar o paciente em seu processo de saúde e doença.

Por conseguinte, entre os fatores associados à cascata do cuidado contínuo às PVHIV, relacionados ao diagnóstico, tratamento e à supressão viral, notam-se desafios a serem enfrentados para garantir cuidado integral a essas pessoas. As evidências científicas apontam que o tratamento promove benefícios à qualidade de vida, como aumento da expectativa de vida, redução da transmissão do vírus, supressão viral, reconstituição imunológica mais robusta, melhores resultados clínicos, redução nas taxas de hospitalizações e de mortalidade^(22,23)^.

Isso também é corroborado nesta pesquisa, onde se evidenciaram associações estatísticas significativas entre pessoas que não estavam em uso da TARV (p=0,000), estavam com carga viral alta (p=0,006) e apresentavam contagem de CD4+ abaixo de 200 cópias/µl (p=0,005), com internações urgentes e óbito. Desse modo, percebe-se que os resultados encontrados vêm para reafirmar a necessidade de implementar tecnologias inovadoras no cuidado, assegurar o compromisso governamental contínuo, envolver a sociedade civil, e, principalmente, estabelecer uma abordagem colaborativa e multidisciplinar no cuidado à saúde das PVHIV^(24)^.

Um estudo brasileiro realizado com 550 PVHIV demonstrou alta prevalência das condições também citadas entre os participantes, como HA (17,89%), DM (7,51%) e DRC (4,83%)^(25)^, embora em níveis inferiores aos encontrados nesta pesquisa. Cabe destacar que, ao analisar as Doenças Crônicas NãoTransmissíveis (DCNT), identificaram-se associações estatísticas que possibilitaram compreender que as PVHIV hospitalizadas no período do estudo com HA, DM e DRC foram as que apresentaram maior risco de óbito e de internação urgente.

Ao analisar os fatores associados à DRC em PVHIV, um estudo brasileiro do tipo caso-controle, realizado com 85 participantes, sendo 17 casos e 68 controles, encontrou que a DRC está associada à HA, aos medicamentos antirretrovirais nefrotóxicos e a pacientes com idade superior a 40 anos^(26)^. Esse fato foi reafirmado em um estudo americano, no qual, além dos fatores citados, o DM também se mostrou uma condição de risco para o desenvolvimento da DRC em PVHIV^(27)^.

A relação entre DM e HIV também vem sendo apontada por outros estudos como uma condição de risco para o agravamento clínico da doença, uma vez que está relacionada à contagem baixa de CD4+, ao uso de inibidores nucleosídeos da transcriptase reversa, inibidores de protease, marcadores inflamatórios circulantes elevados e ao ganho de peso^(28,29)^. Além disso, os estudos indicam que a TARV tem sido associada a um aumento do peso em decorrência do restabelecimento da imunidade das PVHIV^(28)^. De acordo com os achados, constata-se que o DM se manteve como um fator de risco para os participantes, uma vez que aqueles que não referiram ter DM apresentaram um fator de proteção até sete vezes maior para o óbito comparado aos que relataram viver com essa comorbidade.

Assim, levando em consideração a gravidade das DCNT para as PVHIV, faz-se necessária a realização de mais pesquisas que busquem avaliar a qualidade do cuidado a essa população que pode necessitar de hospitalizações, visando uma reflexão acerca do monitoramento dessas comorbidades, da produção de informações, principalmente no que envolve a adequação, benefícios, efeitos adversos, custos de tecnologia, resolutividade e horizontalidade do cuidado. Essas reflexões se consolidam, uma vez que o cuidado às PVHIV deve ser realizado com qualidade, cumplicidade, vínculo e confiança, considerando as barreiras que esses pacientes enfrentam para buscar os serviços de saúde^(30)^.

Ao explorar as sintomatologias apresentadas pelos participantes, percebe-se que febre maior ou igual a 38 graus por tempo maior ou igual a um mês (p=0,016), perda de peso superior a 10% (p=0,013) e astenia (p=0,039) foram marcadores associados à gravidade e ao óbito dos participantes do estudo. Reafirmando esses achados, uma coorte realizada com PVHIV em dois continentes, África e Ásia, demonstrou que febre, perda de peso e astenia são sinais e sintomas clínicos frequentes nos participantes, principalmente naqueles que apresentaram piores prognósticos clínicos da infecção^(31)^. Associado a isso, um estudo realizado na Alemanha com a mesma população enfatiza a importância de o profissional de saúde manter-se alerta para o aumento da febre, visto que ela esteve atrelada principalmente aos pacientes que evoluíram para o óbito no estudo^(32)^.

Nota-se que esses sinais e sintomas podem auxiliar no diagnóstico, monitoramento do agravo e redução dos riscos à saúde desses pacientes em locais onde o rastreamento laboratorial de rotina é limitado devido a restrições financeiras, infraestrutura ou longos tempos de espera por exames. Além disso, a presença desses sinais e sintomas pode servir para orientar a utilização dos testes rápidos no diagnóstico do HIV e, por conseguinte, melhorar a detecção do vírus^(31)^, reduzindo as chances de internações urgentes.

Ao analisar a coinfecção por HIV e HCV, identificou-se que as pessoas admitidas no estudo que apresentaram exames sorológicos de anti-HCV reagentes também foram aquelas que evoluíram a óbito devido ao HIV. Dessa forma, identifica-se que o HCV é frequentemente prevalente em PVHIV^(33,34)^. Ademais, os fatores que levam os pacientes a terem um pior desfecho clínico em decorrência da coinfecção incluem o impacto que o HCV promove na recuperação das células CD4+ dos pacientes em uso da TARV. Aliado a isso, a infecção pelo HIV aumenta as chances desses pacientes desenvolverem fibrose hepática, ressaltando a importância do rastreamento de HCV em pacientes com HIV antes de iniciar a TARV, a fim de impedir que ocorram interações medicamentosas^(33)^.

### Limitações do estudo

Destaca-se o número de participantes da pesquisa, visto que, mesmo atingindo resultados significativos, uma maior população poderia influenciar os resultados e reduzir os intervalos de confiança. Desse modo, ressalta-se a necessidade de maiores investimentos nesse tipo de pesquisa no âmbito hospitalar. Sugere-se a realização de pesquisas multicêntricas para ampliar o olhar em relação à atenção a essa população e possibilitar uma análise comparativa com os achados obtidos.

### Contribuições para a Área

Este manuscrito oferece aos profissionais da saúde, especialmente aos da enfermagem, importantes subsídios para o manejo clínico no atendimento a PVHIV, principalmente no contexto hospitalar. Além disso, o estudo contribui para o direcionamento na prática clínica, seja na organização, planejamento e execução das práticas de cuidado, promovendo também maior autonomia ao profissional.

## CONCLUSÕES

Foi possível identificar três marcadores de prognóstico sociodemográficos e treze clínicos que foram significativamente associados ao risco de agravamento clínico em pessoas hospitalizadas com HIV. Quanto às variáveis sociodemográficas, o sexo masculino foi altamente associado ao óbito e ao risco de hospitalização de forma urgente, enquanto a cor da pele preta/parda apresentou taxas mais elevadas de mortalidade, e a cor branca foi identificada como fator de proteção. Em relação ao nível de escolaridade, as PVHIV com menos de oito anos de estudo apresentaram maior risco de óbito em decorrência dos agravamentos pelo HIV.

No que tange às características clínicas da doença, observou-se que a admissão hospitalar de forma urgente, o diagnóstico descoberto durante a internação hospitalar e a não utilização da TARV são indicadores diretamente associados ao óbito. Com relação aos marcadores biológicos, detectou-se que as pessoas hospitalizadas com carga viral alta e com CD4+ menor que 200 cópias/µl apresentaram maior risco de óbito e de internação urgente, enquanto aquelas com carga viral baixa ou indetectável tiveram alta hospitalar e foram admitidas de forma programada.

Da mesma forma, ter diabetes mellitus, hipertensão arterial e doença renal crônica foram as comorbidades que apresentaram maior risco de agravamento clínico pelo HIV nos participantes. Em relação aos sinais e sintomas associados como fatores de risco para o óbito e internações urgentes, a febre maior ou igual a 38°C por tempo maior ou igual a um mês, perda de peso superior a 10% e astenia estão entre os indicadores de pior prognóstico. Quanto aos exames sorológicos realizados pelos participantes, a coinfecção por HCV foi o único indicador que se destacou como maior fator de risco.
